# Potential benefits of climate change on navigation in the northern sea route by 2050

**DOI:** 10.1038/s41598-024-53308-5

**Published:** 2024-02-02

**Authors:** Mohamed Rami Mahmoud, Mahmoud Roushdi, Mostafa Aboelkhear

**Affiliations:** Environment and Climate Changes Research Institute, National Water Research Center, Cairo, Egypt

**Keywords:** Climate sciences, Environmental sciences

## Abstract

Climate change has been inducing a continuous increase in temperatures within the Arctic region, consequently leading to an escalation in the rates of Arctic ice depletion. These changes have profound implications for navigation along the Arctic Northern Sea Route (NSR). However, access to the NSR is constrained to specific temporal intervals when the sea ice thickness reaches a threshold that permits safe passage of ships. This research employs climate change model simulations and the Polar Operational Limit Assessment Risk Indexing System framework to investigate the navigational feasibility of diverse ship types along NSR during the calendar years 2030, 2040, and 2050, under SSP2-4.5 and SSP5-8.5 scenarios. Different ship categories were analyzed within the context of these two scenarios. Results indicate considerable variation in the navigability of different ship categories across different scenarios and years. In general, polar ships demonstrate a higher navigational potential throughout most of the year, while pleasure crafts are constrained to specific periods. These findings bear significant implications for the future of shipping along the NSR. As Arctic ice continues to melt, NSR is anticipated to become more accessible to ships, albeit with navigational availability remaining contingent on the ship category and seasonal considerations.

## Introduction

More than 80% of commodities exchanged in the international market are transported via maritime routes, constituting approximately 40% of the overall global economy^1^. Accordingly, container shipping was valued at around US$14 trillion in 2019^2^. Maritime trade, encompassing the exchange of goods, and transportation, involving the movement of individuals, provide vital support to all sectors of the global economy, exerting significant influence on economic expansion, social advancements, mitigating health hazards, and contributing to poverty reduction^1^.

Interoceanic canals play a crucial role in facilitating the movement of maritime commerce across global networks, making them key indicators of global trade and shipping operations. When considering the network-centric perspective, hub ports exhibit common characteristics in terms of their navigational accessibility and connectivity within shipping networks. However, the strategic importance of hub ports in global shipping networks depends on their proximity to major maritime passages. In the highly competitive and expanding global market, the objective is to discover new routes and untapped markets to maximize economic benefits and promote trade activities^3^.

Due to the accelerated ice and ice melt in the Arctic region caused by recent global warming trends, NSR has become a focal point for examination^4^. It is highly likely that the Arctic will experience more pronounced surface warming compared to the global average during the twenty-first century^5^. This warming is anticipated to result in an intensified polar water cycle, leading to an increase in average precipitation. Furthermore, it is expected that the intensity of precipitation will strengthen, with a higher likelihood of it being in the form of rainfall rather than snowfall^6^. According to the IPCC's sixth assessment report, all Shared Socioeconomic Pathway (SSP) scenarios indicate that the minimum annual Arctic sea ice area will drop below 1 million Km^2^ at least once before 2050^6^. This is primarily due to the Arctic region experiencing a warming rate at least twice as fast as the global on average^7^. Several experts have projected that the summer sea ice cover in the NSR will experience a significant reduction under the assumption of ongoing melt rates of the Polar Ice Cap. This could potentially lead to a state of substantial ice-free conditions by the year 2050^8^. This alarm was first published by the Arctic Council in 2004, since all climate models agree that climate change will lead to Arctic warming^9^. Tanskanen^10^ reported that sea-surface temperatures in the Arctic Ocean have been increasing, particularly in the Chukchi Sea. August temperatures in this region rose at a rate of 0.7 °C per decade from 1982 to 2017. Lei et al.^11^ and Wang et al.,^12^ investigated the changes in sea ice conditions along the Arctic Northeast Passage over the past four decades. Their analysis of remote sensing data from 1979 to 2012 reveals a significant decrease in Arctic ice thickness, resulting in a longer open period for navigation. This increased accessibility of Arctic sea routes has important implications for maritime transport and trade. A study by Min et al.^13^ examined the long-term accessibility of the trans-Arctic region for maritime transport. They found that sea ice has decreased significantly over the past four decades, making the Arctic Ocean more accessible for shipping. However, navigability is still limited by sea ice, and the potential for year-round shipping remains uncertain. The North Atlantic Sailing Route has seen an increase in navigable days, particularly for Open Water and Polar Class 6 ships, despite interannual and interdecadal variability. The systematic assessment of Arctic navigability serves as a valuable reference for projecting future trans-Arctic shipping routes. However, it is important to note that the rapid loss of sea ice in polar regions is creating new opportunities^14^, but it is also amplifying the risks associated with shipping and other economic activities in these regions. NSR is facing complex scientific, economic, and legal challenges^15^, in addition to numerous navigational and operational challenges^16^. Its remoteness complicates rescue efforts, and environmental dangers like ice, storms, and extreme temperatures pose risks to safe travel^17,18^. The increased occurrence of sea fog over areas with retreating sea ice poses a new challenge for Arctic navigation, causing significant delays in shipping routes previously underestimated^19^. Environmentally, The NSR risks shipping accidents, oil spills, air pollution, and other unexpected consequences^20^. Thus, in case of NSR is opening up, it is needed to start thinking critically about the legal, environmental, social and geopolitical implications^21^.

In the Arctic, navigational technology faces significant limitations. The effectiveness of the Global Positioning System (GPS) is reduced due to limited satellite coverage at high latitudes. Additionally, the magnetic compass becomes unreliable in finding true north, and the gyrocompass also struggles with accuracy in this region^22^. In addition, radio communications from ship-to-shore stations is more challenging due to line-of-sight and distance variables to operational shore stations’ Medium Frequency (MF) and High Frequency (HF); thus, success is variable^23^. Hence, Satellite communication, particularly through the Global Maritime Distress and Safety System (GMDSS), is essential for effective ship-to-shore communication, using satellite and radio technologies for messaging.

The varying depths along the NSR and the infrastructure of its ports, affected by ice floes, challenge reliable global supply chains^24^. Additionally, the salvage and recovery infrastructure in the Arctic is underdeveloped, making operations costly and logistically difficult^25^. Maritime security is crucial for Arctic nations due to rising interest in the region's resources and strategic location, leading to territorial disputes. Countries like Canada are improving their military capabilities for Arctic patrol, but the vastness of the region and the nature of sea-based threats demand enhanced strategies and military readiness in both land and sea domains^15,26^. Accordingly, collaborative diplomacy is essential to prevent disputes from escalating and to ensure that the Arctic remains a region of peaceful cooperation.

This research focuses on the existence of reliable navigation routes. Hence, the objective of this study is to assess the anticipated variations in Arctic ice cover thickness within the context of climate change, along with its consequent impacts on the navigational activities of vessels and tankers traversing the Northern Sea Route. Antecedent scholarly investigations have provided evidence that accessing this specific route is confined to specific temporal windows within the annual cycle, while remaining inaccessible for the remainder of the period.

## Methods

Recent endeavors in maritime research have focused on exploring various navigational paths through the Arctic region^14,27–30^. However, these investigations have predominantly been constrained to a limited scope, typically encompassing only one or two types of vessels. A significant gap in these studies is the lack of comprehensive analysis concerning the diversity of ship categories and the associated risks they face throughout the year in Arctic waters. Furthermore, there is a discernible need for a robust and detailed methodological framework capable of encompassing a wide spectrum of vessel types and systematically assessing their respective navigational risks in the Arctic region.

### Study area

**Figure 1:**
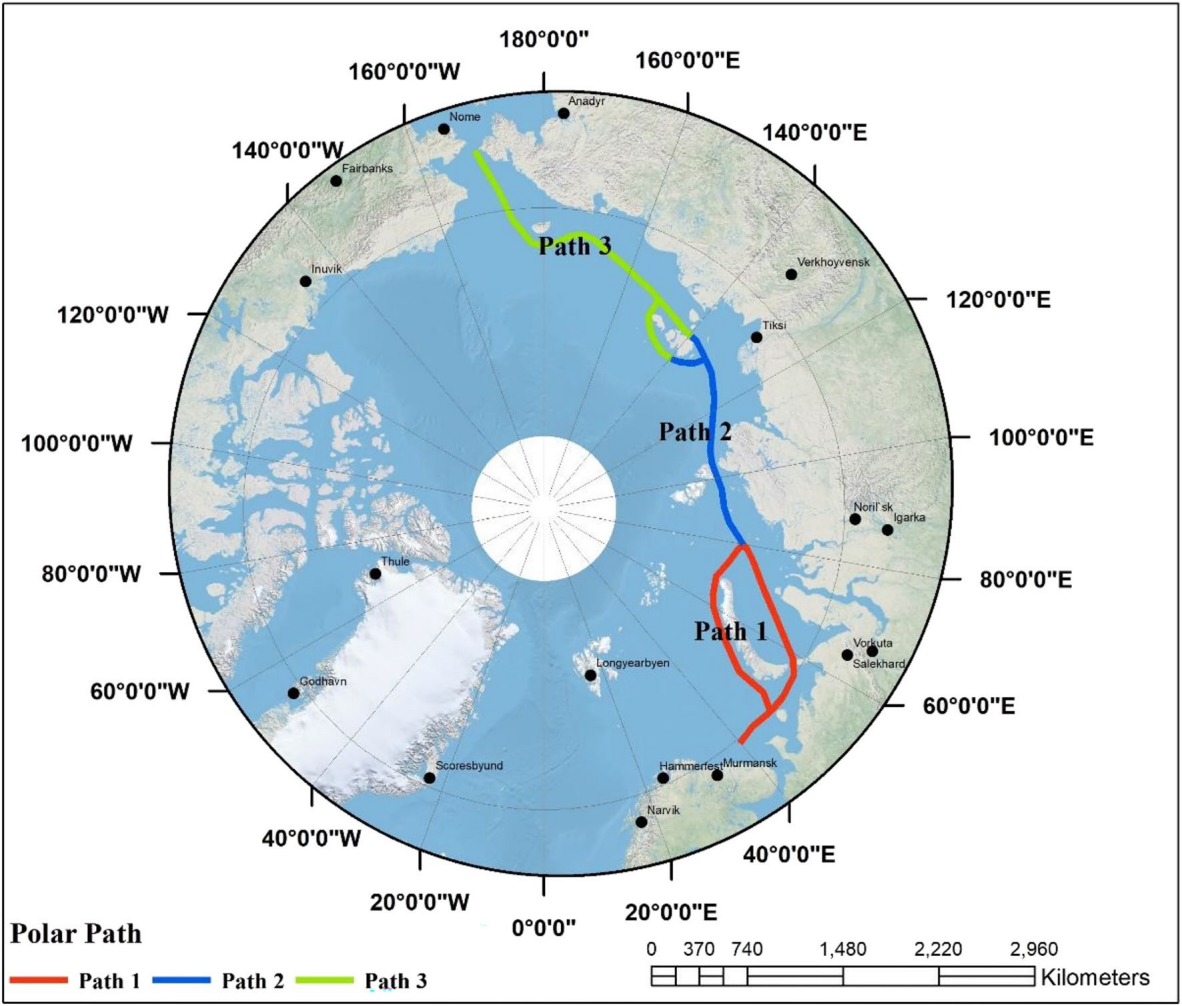
The Arctic Northern Sea Route (NSR) (using ArcGIS 10.8.1 desktop, www.esri.com).

The study area holds significant global strategic importance owing to its commanding geographical location, affording extensive panoramic views that span across multiple countries on various continents. The region notably encompasses a critical maritime route. It has been demarcated into three discrete climatic regions. Path 1 is situated within the first climatic region, encompassing latitudes ranging from 70° to 76° North and longitudes spanning from 40° to 80° East. Path 2 is located within the second climatic region, spanning latitudes of 76° to 75° North and longitudes of 80° to 140° East. Concurrently, Path 3 resides within the third climatic region, extending across latitudes from 75° to 66° North and longitudes from 140° to − 170° East, (Fig. 1). The remarkable strategic significance of this study area, combined with its unique climatic divisions, makes it a compelling subject for comprehensive academic exploration and analysis.

### Ice cover thickness and mean temperature projection in the North Pole

The Coupled Model Intercomparison Project Phase 6 (CMIP6) models exhibit improved capability in simulating the sensitivity of Arctic sea ice area to anthropogenic CO_2_ emissions, leading to a more accurate representation of the temporal evolution of Arctic sea ice loss, as observed by satellite^31^. The capacity to model ice-sheet processes has undergone significant enhancement since the Fifth Assessment Report (AR5). As a result, the representation of fundamental processes related to surface-mass balance and retreat of the grounding-line, in the absence of instabilities, is deemed to have medium confidence. Nevertheless, simulations of ice-sheet instabilities, ice-shelf disintegration, and basal melting still carry low confidence due to their high susceptibility to uncertain oceanic forces, boundary conditions, and parameters^6^. In this study, the Community Earth System Model (CESM) was employed to predict the mean temperature and the ice thickness in the Arctic, which affects navigation routes. Additionally, the model was used alongside other models to predict and evaluate climatic conditions^32–35^.

The CESM is a coupled climate model that simulates the Earth's atmosphere, ocean, land surface, sea ice, and a central coupled component. It is one of the products of the National Science Foundation (NSF) and is based at the National Center for Atmospheric Research (NCAR) in Boulder, Colorado, USA. The latest version of the model, CESM2, which was used in this study, includes many new technological and scientific capabilities, such as a more realistic representation of the evolving ice sheets in Greenland, detailed modeling of crop interactions with the Earth system, improved representation of clouds and precipitation, and the addition of wind-driven wave modeling on the model's ocean surface^36^. A large set of data was used in simulations, including pre-industrial conditions in the simulation period period from 1850 to 2014. The CESM is a distinguished model in predicting ice thickness, as reported by Mudryk et al.,^37^.

During the study, monthly data for North Arctic Sea Ice thickness was used from 2020 to 2050, using the SSP2-4.5 climate scenario, which is the expected average scenario, as well as the pessimistic scenario SSP5-8.5.

#### Operating ships in the arctic region

The Polar Class (PC) designation is a standardized system developed by the International Association of Classification Societies (IACS) to classify ships based on their ability to operate in polar waters. The PC designation ranges from PC1 for year-round operation in all polar waters to PC7 for operation in thin first-year ice in summer and autumn of the first year^38^, Table 1. There are also non-polar ship classes, such as Type IA, Type IB, and Type IC ships, that can operate in specific periods of the year and are designed to operate in open water or in ice conditions less severe than those included in PC^39^. These classifications help to ensure that ships are designed and built to operate safely and efficiently in the challenging ice conditions of the Arctic region.

**Table 1:** Categories of Polar Ships and their Operating Conditions.

Polar class	Ice description (based on world meteorological organization sea ice nomenclature)	
	Time period	Areas of operation
PC1	Year-round	All Polar waters
PC2		Moderate multi-year ice conditions
PC3		Second-year ice which may include multi-year ice inclusions
PC4		Thick first-year ice which may include old ice inclusions
PC5		Medium first-year ice which may include old ice inclusions
PC6	Summer/autumn	Medium first-year ice which may include old ice inclusions
PC7		Thin first-year ice which may include old ice inclusion

In 2022, a total of 2994 voyages were conducted through the NSR by 314 vessels. CHNL^40^ mentioned that the highest number of voyages occurred in August and September, when the ice thickness was at its minimum, reaching up to 500 voyages. On the other hand, during the summer and spring seasons, the number of voyages was at its lowest, averaging around 150 voyages.

#### Identifying the ice hazard

The risk of polar navigation depends on the thickness of the ice and the type of ship used. The capability of each ship type to navigate through ice varies according to its equipment, class, or rank. The highest-ranked and most well-equipped ship for polar navigation is the PC1 ship, followed by PC2, PC3, and so on up to PC7. Ships of the non-polar classes IA, IB, and IC are less equipped than Polar Class ships and thus face greater difficulties navigating through polar regions. Pleasure craft and other non-polar ship types fall under the No Ice Class category. The level of risk that ships face depends on their type, equipment, and the type (thickness) of ice. The lowest risk falls on fully equipped PC1 polar ships, followed by PC2, PC3, up to PC7, with higher levels of risk for non-polar IA, IB, IC ships, and the highest risk level for pleasure craft. The highest risk occurs with heavy multi-year ice, while the lowest risk is associated with new ice^41^.

### The polar operational limit assessment risk indexing system (POLARIS)

The Polar Code, which came into force on January 1, 2017, is a mandatory requirement for all new and existing ships operating within the International Maritime Organization (IMO)-defined boundaries of Arctic waters and the Antarctic area, whether on domestic or international voyages. The Polar Code requires an approved methodology for determining the operational limitations of a ship to ensure its safe navigation through these regions^42^. The Polar Operational Limit Assessment Risk Indexing System (POLARIS) has been acknowledged as an acceptable methodology for such purposes and is therefore considered highly relevant for all ships sailing within the relevant areas^43^. POLARIS is designed to provide a methodology for assessing the ship-specific capabilities and limitations under different ice regimes and operational modes. The application of POLARIS offers numerous advantages and can be employed for various purposes. For example, POLARIS can be used as a risk assessment tool for voyage planning, either aboard or ashore. It can also be used at the design stage for selecting the appropriate ice class of the vessel. Ensuring an acceptable level of risk while operating in diverse ice regimes and types of operation is crucial^43^. Therefore, while planning a voyage or an operation, it is necessary to consider the structural capabilities of the ship, its characteristics, type of operation, as well as current and anticipated ice conditions.

POLARIS evaluates the risks associated with ice conditions specific to the ship's ice class through employment of a Risk Indexing Value (RIV) indicator that is assigned to the ship based on its ice class and ice types^44^. The use of POLARIS can help to ensure the safe navigation of ships in polar regions. It is a valuable tool for shipowners, operators, and regulators to assess the risks associated with operating in these challenging environments^43^.

The future ice thickness obtained from the CESM2 climate model is converted into a risk index through the Risk Index Outcome (RIO) factor. The RIO is allocated based on the ship's ice class and the type of ice. The ice types are classified into 12 categories based on their thickness.

Ice thickness is converted to the RIO risk coefficient for a region (section) of 100 km using Eq. (1):

1 $${{\varvec{R}}{\varvec{I}}{\varvec{O}}}_{{\varvec{c}}}=\sum_{{\varvec{i}}=1}^{{\varvec{i}}=12}{{\varvec{n}}}_{{\varvec{i}}}\times {\varvec{R}}{\varvec{I}}{{\varvec{V}}}_{{\varvec{i}}.{\varvec{c}}}$$

where RIO_c_ = Risk Index Outcome factor for ship's ice class c (value ranges from -80 to + 30), n_i_ = the number of 10’s km with the same ice thickness i, RIV_i,c_ = the corresponding Risk Index Values based on the ship's ice class c and ice thickness i, ranging from zero (no ice) to over three meters for multi-year ice, obtained from Table 2

**Table 2:**
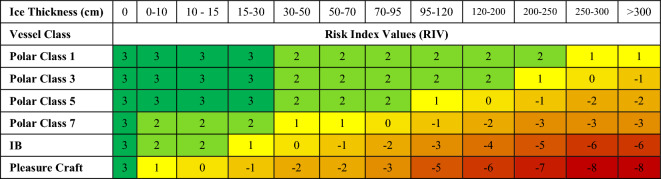
Risk index values for different types of ice and ships.

Table 2 summarizes ice thickness and Risk Index Value for each type of ice and studied ships^45,46^.

**Figure 2:**
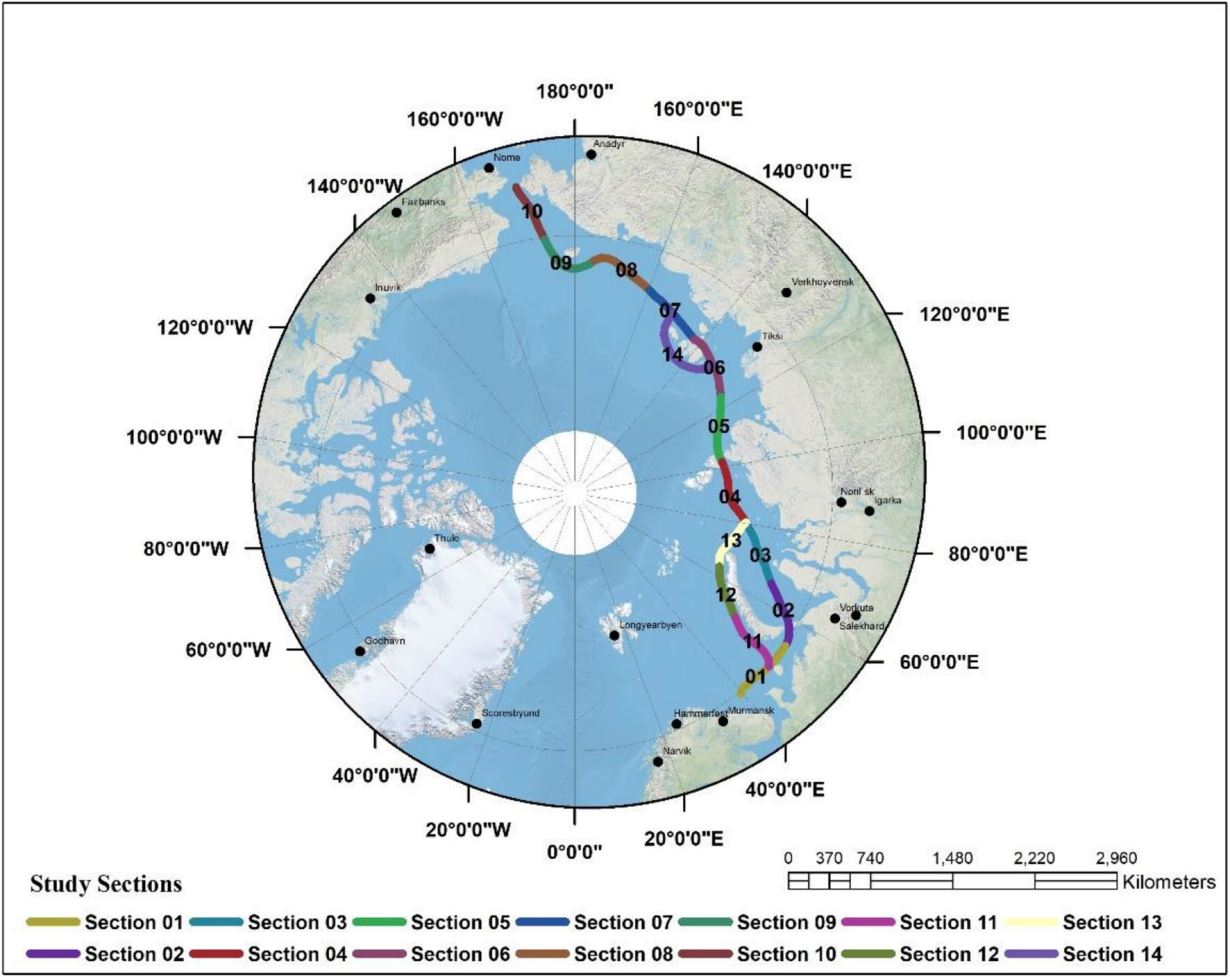
The fourteen sections of the navigational route. (using ArcGIS 10.8.1 desktop, www.esri.com).

The POLARIS framework considers positive RIOs to be indicative of normal operating conditions and an acceptable level of risk for vessel navigation. Negative RIOs, on the other hand, are considered to be unacceptable risk indicators and should be avoided. The "go or no-go" terminology is used to express positive and negative values, based on the type of ice and vessel category. Positive values ("go") indicate that the vessel is capable of passing and navigating, while negative values ("no-go") indicate that the vessel is unable to pass and navigate. To calculate the probability of vessel passage through the navigational route in different months up to the year 2050, it was necessary to divide the route into a series of sections and conduct analyses for each section separately. The navigational route was therefore divided into 14 sections, each with an approximate length of 100 km. The RIO has been determined for the 14 sections, (Fig. 2). All sections, representing the entire path, were combined to express the navigability of ships through the navigational path for each month separately for the calendar years 2030, 2040, and 2050.

## Results

### Projection of temperature and sea ice thickness

**Figure 3:**
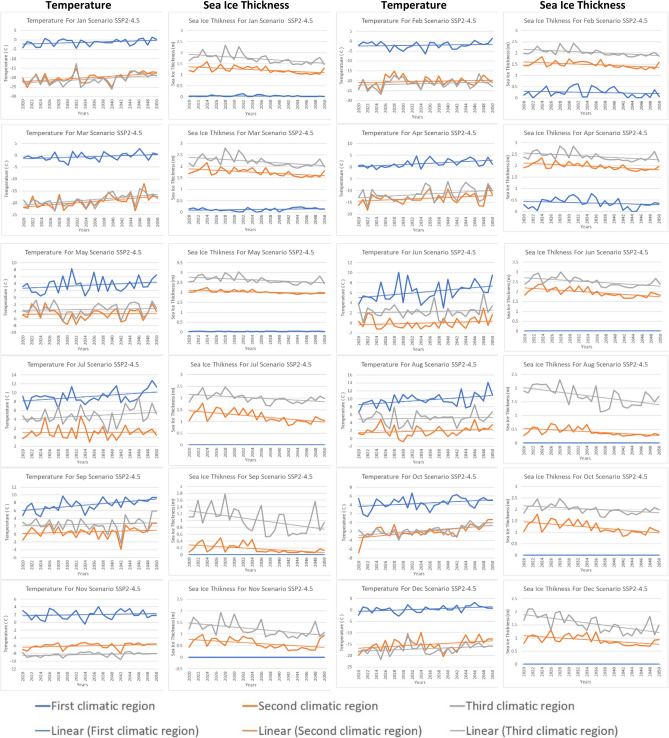
Projection of temperature and sea ice thickness using SSP2-4.5.

**Figure 4:**
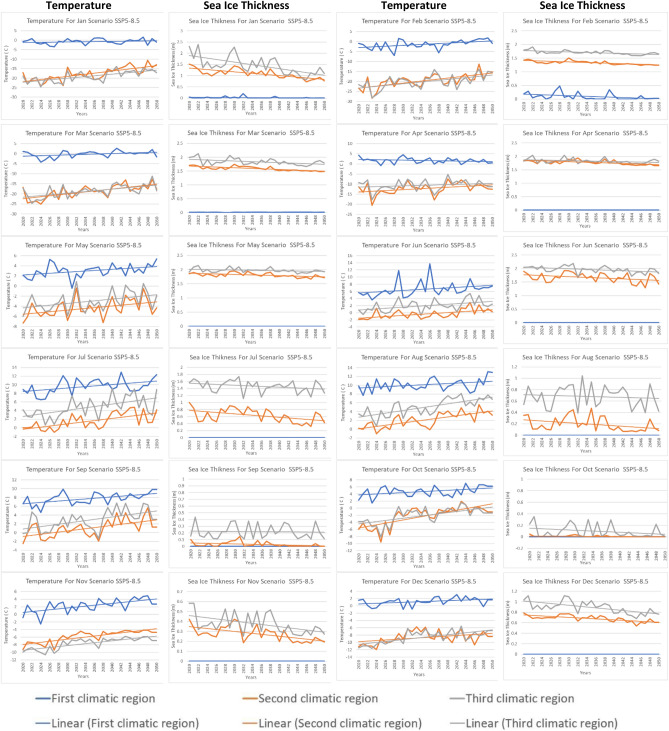
Projection of temperature and sea ice thickness using SSP5-8.5.

Variations in temperature and sea ice thickness have a significant impact on the movement of polar ships. Polar regions are characterized by harsh environments with extreme cold temperatures and extensive sea ice coverage^47^. The minimum Arctic ice thickness occurs annually in September and has exhibited a significant decline throughout the satellite era spanning the past decades. Additionally, notable fluctuations in ice thickness occur both on a year-to-year and month-to-month basis, yet the specific mechanisms driving this variability remain uncertain^48^. Figure 3 shows the monthly average predictions of sea ice thickness for the three climatic regions from 2020 to 2050 for the SSP2-4.5 scenarios. It indicates that the ice thickness for the first climatic region is the lowest of all months, approaching zero in August, September, October, November, and December. In contrast, the ice thickness for the second and third climatic regions ranges from 1.5 to 3 m in most months during the 2020–2030 period. Additionally, Fig. 4 shows the monthly average predictions of sea ice thickness for the three climatic regions from 2020 to 2050 for the pessimistic SSP5-8.5 scenarios. It indicates that the ice thickness for the first climatic region is the lowest of all months, approaching zero in all months. In contrast, the ice thickness for the second and third climatic regions ranges from 0.5 to 2 m in most months during the 2020–2030 period. Overall, sea ice thickness is expected to decrease significantly until it reaches its lowest level in 2050 for both scenarios.

The first climatic region experiences significant variations in temperature changes throughout the year under different scenarios. Under the SSP2-4.5 scenario, April has the highest annual temperature increase of about 0.051 °C/year, followed by May with an annual increase of about 0.037 °C/year and June with an annual increase of about 0.042 °C/year. July and August have similar annual temperature increases of about 0.044 °C/year and 0.047 °C/year, respectively. October has the lowest annual temperature increase of about 0.045 °C/year, and December has a moderate annual temperature increase of about 0.039 °C/year. In the SSP5-8.5 scenario, June has the highest annual temperature increase of about 0.063 °C/year, followed closely by July with an annual increase of about 0.060 °C/year. September has the same annual temperature increase as July, at about 0.063 °C/year. October has a moderate annual temperature increase of about 0.058 °C/year, while August has a slightly lower annual temperature increase of about 0.0518 °C/year. March and May have moderate annual temperature increases of about 0.064 °C/year and 0.0557 °C/year, respectively.

In Region 2, temperature and sea ice thickness vary across different months and scenarios. In April, under the SSP2-4.5 scenario, the temperature shows a gradual yearly increase of approximately 0.031 °C/year, while the sea ice thickness experiences a yearly decrease of around 0.010 m/year. For June, the temperature increase slows down to approximately 0.032°C/year under the same SSP2-4.5 scenario, and the sea ice thickness experiences a slightly higher yearly decrease of about 0.015 m/year. In October, the temperature under the SSP2-4.5 scenario shows a significant yearly increase of approximately 0.039 °C/year, while the sea ice thickness continues to decrease at a rate of around 0.0164 m/year. When considering the SSP5-8.5 scenario in June, the temperature experiences a yearly increase of about 0.052 °C/year, slightly higher than the SSP2-4.5 scenario. However, the sea ice thickness decrease is less severe under the SSP5-8.5 scenario, with a yearly reduction of around 0.007 m/year. Overall, these observations suggest that Region 2 experiences a varying rate of temperature increase and sea ice thickness decrease throughout the year, with the SSP2-4.5 scenario generally leading to higher temperature increases and more significant sea ice thickness reductions compared to the SSP5-8.5 scenario.

In Region 3, different months and scenarios exhibit distinct patterns in temperature and sea ice thickness changes. In February, under the SSP2-4.5 scenario, the temperature shows a yearly increase of approximately 0.032 °C/year, while the sea ice thickness experiences a yearly decrease of around 0.01 m/year. Moving to July, the temperature increase remains relatively consistent with a yearly rise of about 0.042 °C/year under the same SSP2-4.5 scenario. Similarly, the sea ice thickness experiences a slightly higher yearly decrease of approximately 0.01 m/year. In October, the temperature under the SSP2-4.5 scenario displays a somewhat higher yearly increase of about 0.040 °C/year, while the sea ice thickness continues to decrease at a rate of around 0.01 m per year. Finally, in December, the temperature increase is slightly lower compared to October, with a yearly rise of approximately 0.061 °C/year under the SSP2-4.5 scenario. However, December experiences a more significant yearly decrease in sea ice thickness, around 0.022 m/year, compared to other months under the same scenario.

#### Projection of precipitation

**Figure 5:**
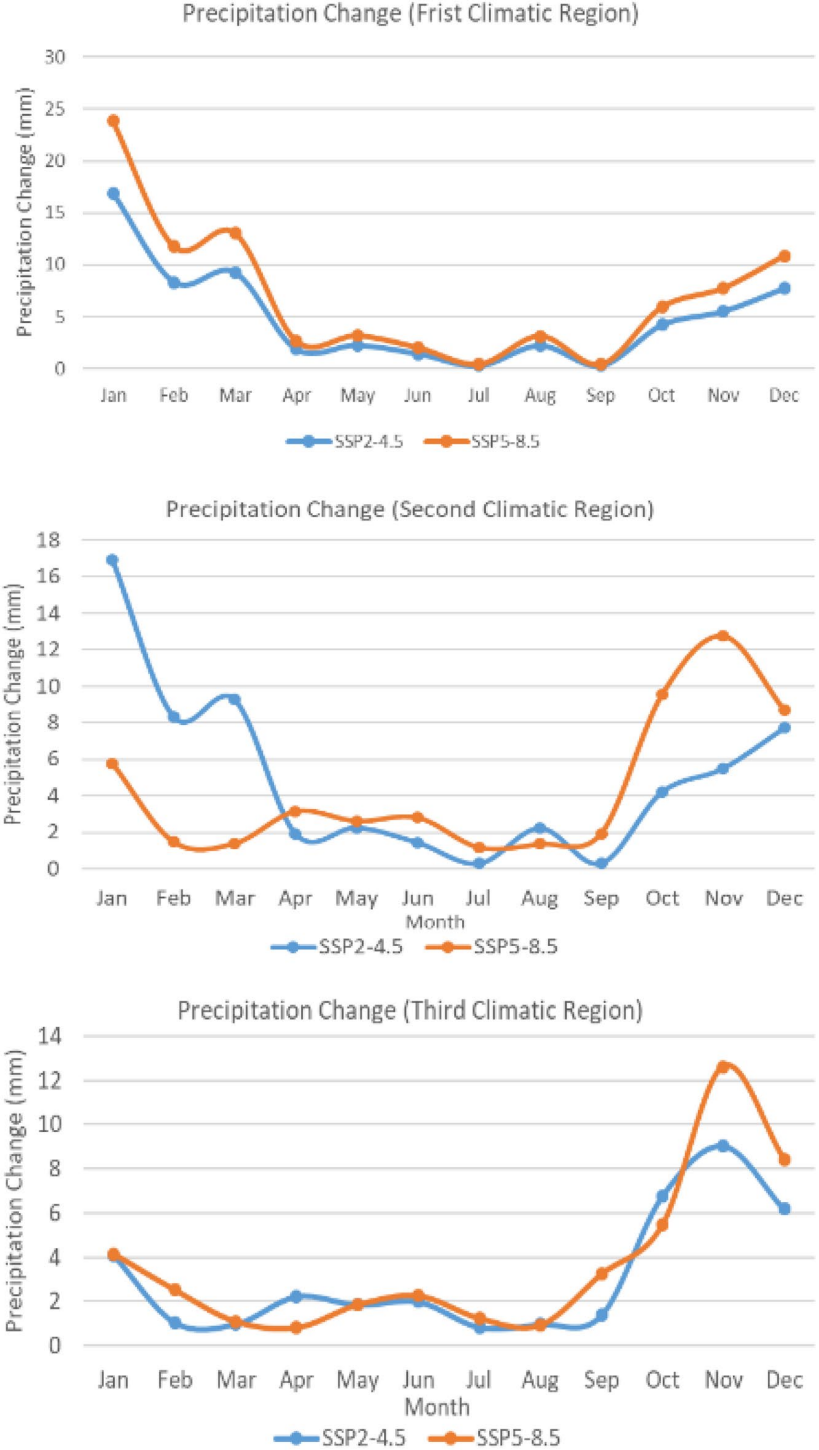
Projection of precipitation change (2050–2020) under SSP2-4.5 and SSP5-8.5.

Figure 5 illustrates projected changes in monthly precipitation for three climatic regions along the NSR by the year 2050, using SSP2-4.5 and SSP5-8.5. Climate projections generally indicate a slight increase in precipitation for each of the three climatic regions in 2050. The projections in the first climatic region indicate that, regardless of the scenario, summer months could see a decrease in precipitation change, with the lowest values in July, while winter months would see the highest precipitation change, particularly in January to March. In the second climatic region, the two scenarios predict a sharp increase in precipitation, especially from October to March. The projections imply minimal increases during summer, with no alteration in July and September under the SSP2-4.5 scenario. The projections for the third climatic region align with a comparable pattern, wherein SSP2-4.5 and SSP5-8.5 anticipate reduced precipitation changes in summer, and the peak increment is projected to occur in November.

#### Projected Navigation Feasibility of the NSR

**Figure 6:**
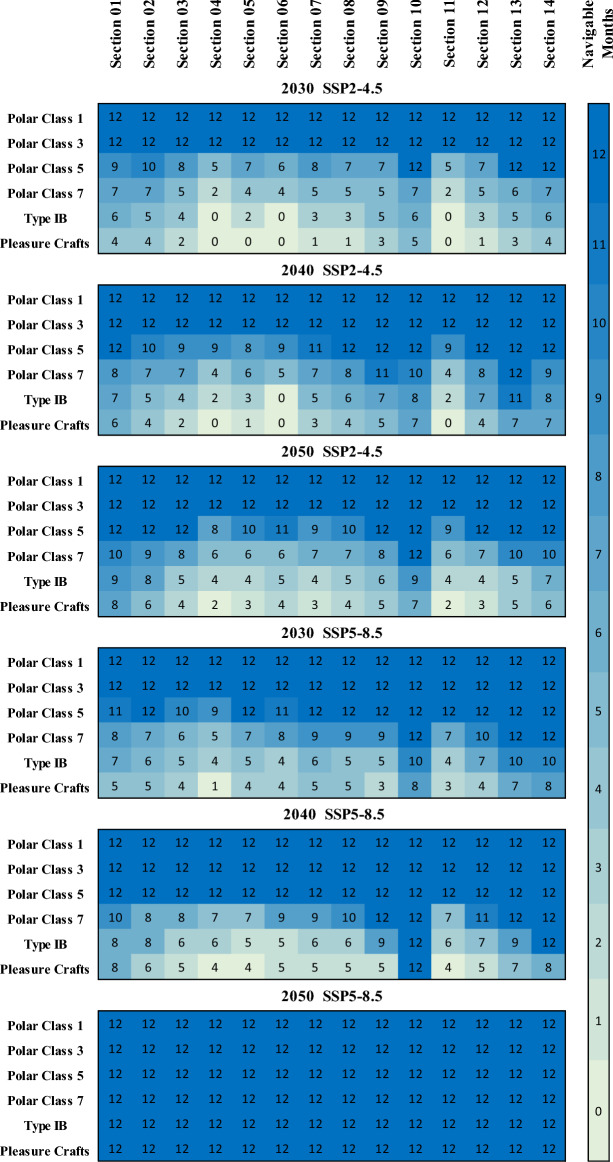
Assessment of monthly navigational feasibility by section for various ship types under SSP2-4.5 and SSP5-8.5.

The reduction in sea ice thickness bears significant implications for transpolar shipping operations, primarily influencing the suitability of vessels capable of navigating ice-covered waters. The diminishing sea ice permits the utilization of polar-class vessels with lower ice class designations, leading to reduced fuel consumption and emissions^8^. This study investigated the impact of climate change on the navigation feasibility of the NSR for different ship categories. Figure 6 presents a detailed analysis for each of the 14 sections constituting the navigation route. It delineates the number of months per annum during which a specific type of vessel can navigate through a given segment of the route, as per the projected climate change scenarios for the years 2030, 2040, and 2050. The examined ship categories encompass Polar class 1, Polar class 3, Polar class 5, Polar class 7, Type IB, and Pleasure crafts. Within the SSP2-4.5 scenario in 2030, Polar class 1 and Polar class 3 vessels consistently demonstrate availability throughout the year. Polar class 5 ships initially experience unavailability in the first seven months, with subsequent accessibility from August to December. Conversely, Polar class 7 ships are unavailable for ten months and will only be accessed in September and October. Type IB ships and Pleasure crafts uniformly remain unavailable throughout the year. For the SSP5-8.5 scenario in 2030, Polar class 1 and Polar class 3 ships maintain continuous availability. Polar class 5 ships operate during the first trimester, followed by inoperability from April to June, with functionality from July to December. Polar class 7 ships lack availability during the first trimester but subsequently become accessible from August to December. Type IB ships exhibit limited availability, excluding August to December. Pleasure crafts remain nonoperational in the initial nine months but become viable in September.

Transitioning to the 2040 scenario, both SSP2-4.5 and SSP5-8.5 scenarios indicate uninterrupted availability of Polar class 1 and Polar class 3 vessels throughout the year. Polar class 5 ships, except in the first trimester under SSP2-4.5, demonstrate continual operability, while under SSP5-8.5, they maintain viability throughout the year. Polar class 7 ships, similar to the previous scenario, are unavailable during the first trimester but subsequently become accessible. Type IB ships consistently exhibit unavailability throughout the year, except for August and September in both scenarios. Pleasure crafts show non-availability throughout the year, except September through December in SSP5-8.5.

Considering the 2050, ship availability patterns mirror those of the 2040. Polar class 1 and Polar class 3 vessels remain consistently accessible throughout the year in both scenarios. Polar class 5 ships are operational, except for the initial three months under SSP2-4.5. Polar class 7 ships exhibit perpetual availability under SSP5-8.5 only. Type IB ships maintain year-round availability under SSP5-4.5 for August to November. Pleasure crafts demonstrate accessibility in under SSP2-8.5. These results concur with the findings of Chen et al.,^49^, which suggest that Polar class 6 ships, falling between Polar class 5 and Polar class 7, would be capable of traversing the NSR from August to December during 2021–2025 and from July to December during 2026–2050.

## Discussion and conclusion

This investigation provides significant contributions to the scholarly comprehension of the ramifications of climate change on Arctic navigation, extending its relevance to the formulation of environmental policies and the operational strategies of maritime industries. It offers a valuable predictive framework for elucidating future trajectories in Arctic shipping routes. Furthermore, this study lays the groundwork for subsequent research aimed at optimizing maritime routes, incorporating a comprehensive consideration of the diverse requisites of various maritime sectors. Additionally, the inclusion of an array of ship categories in this analysis facilitates a deeper exploration into the economic dimensions of Arctic navigation, presenting opportunities for further detailed economic assessments. Moreover, this research distinctly advances our understanding by conducting analyses for each of the 14 sections of the Arctic routes, tailored to accommodate varying types of ships. This segmentation of the route into discrete sections and their subsequent individual analysis is a novel approach not previously explored in existing studies. This detailed breakdown enables future research to meticulously examine each section in relation to different ship categories, thereby offering a more granular and precise understanding of Arctic navigation. Such an approach has the potential to yield unprecedented insights into the interaction between specific route sections and various ship types, facilitating a more targeted and efficient assessment of navigational strategies and risks in the Arctic context.

The consequences of climate change on the NSR are of substantial and multifaceted significance. The diminishing sea ice in these regions has ushered in novel shipping opportunities, albeit with attendant complexities. Polar vessels are particularly susceptible to temperature fluctuations and variations in sea ice thickness, which introduce risks and difficulties in the demanding Arctic and Antarctic environments. Temperature fluctuations influence the melting and formation of sea ice, consequently impacting navigational conditions, either facilitating or hindering maritime transit. Ships operating in polar regions must be attuned to these environmental fluctuations and adapt their routes accordingly, as thicker ice necessitates specialized navigation capabilities, whereas thinner ice conceals latent hazards.

Furthermore, the composition of Arctic Sea ice has undergone a noteworthy transformation, shifting from predominantly thick multi-year ice to younger and thinner seasonal ice. The likelihood of an ice-free Arctic summer increases in tandem with global warming. Under the scenario of stabilizing global warming at 1.5°C, there is an approximate 2% chance of an ice-free summer occurring in any given year, with this probability rising to 19–34 percent at 2 °C. Some computational models even project that the Arctic Ocean could become seasonally ice-free within the coming decades^10^.

The observations generated by this study align with the findings of Chalecki^9^, who previously reported that contemporary models suggest a transition from gradual ice thinning to rapid ice loss, potentially resulting in a virtually ice-free Arctic during the summer by 2040, an earlier timeframe than previously anticipated. Additionally, both Ravindran et al.,^50^ and Notz and Community, 2020^31^ have indicated the likelihood of an ice-free Arctic surface during September occurring prior to 2050: a substantial reduction or absence of sea ice cover during that specific period.

This study examines the accessibility of the NSR to different ship categories under two climate change scenarios SSP2-4.5 and SSP5-8.5 for 50 years. The ship categories considered are Polar class 1, Polar class 3, Polar class 5, Polar class 7, Type IB, and Pleasure crafts. Polar class 1 and Polar class 3 vessels consistently maintain accessibility throughout the year for both scenarios. Polar class 5 vessels experience fluctuations in their availability, initially marked by unavailability followed by subsequent periods of accessibility. Polar class 7 ships predominantly exhibit unavailability during specific trimesters but subsequently become accessible during other months for SSP2-4.5 scenario. Type IB ships and Pleasure crafts consistently register unavailability, with sporadic windows of accessibility in particular months for SSP2-4.5 scenario, while they ensure continuous accessibility throughout the year for SSP5-8.5. These findings yield valuable insights for the strategic planning of shipping route availability and the potential allocation of resources along these routes.

Lin et al.,^51^ delineated the primary challenges and hazards confronting ships traversing Arctic shipping routes, encompassing:

- Factors related to low temperature
- Factors related to sea ice
- Factors related to poor visibility
- Factors related to communication
- Lack of infrastructure
- Lack of navigational experience
- Lack of historical data
- High risk of collision
- Complex navigational conditions

These factors have been scrutinized in the context of the climate change scenarios delineated in the study. Observations indicate that reduced temperatures could potentially impair crew performance, attenuating their responsiveness to external conditions and decreasing precipitation, as corroborated by Zhang et al.^52^. However, an increase in ambient temperatures along the selected route could potentially ameliorate these risks.

In the context of sea ice factors, Eq. (1), which quantifies navigational risks for ships, has been employed in this study. This equation encapsulates the hazards associated with floating ice, as documented by Li et al.^53^ and Xu et al.^54^. The Arctic region is characterized by poor visibility factors, primarily attributable to sea ice and snow, as noted by Goerlandt et al.^55^. Projections under both climate change scenarios indicate a slight increase in precipitation over the recommended path (Fig. 5), suggesting that the risk associated with poor visibility is unlikely to diminish.

Communication deficiencies, one of the leading causes of ship collision in Arctic shipping routes^54,56^, can be attributed to the limitations of mobile data collection equipment, such as unmanned aerial vehicles (UAVs), under low-temperature conditions^51,57^. These devices encounter operational restrictions, resulting in constrained data acquisition measures. However, under the projected climate change scenario, a marginal increase in temperature could potentially alleviate these constraints. The anticipated rise in ambient temperatures along the selected trajectory could serve as a mitigating factor against these risks.

It is also noteworthy to mention that the mobility of ice floes, which are persistently influenced by wind patterns, poses a significant challenge for ship-based communication^58^. This dynamic nature of the ice floes further exacerbates the communication difficulties experienced in these environments.

In relation to the potential hazards associated with the absence of infrastructure, lack of navigational experience, and deficiency of historical data, it is hypothesized that these risks will likely diminish in correlation with an increase in maritime voyages, as predicted by climate change models. This assertion is predicated on the assumption that increased frequency of ship trips will contribute to the accumulation of experience and data, thereby mitigating these identified risks. Further empirical investigation is warranted to substantiate this hypothesis.

Incidents involving collisions between icebreakers and the vessels they escort represent the most frequently reported accidents in the context of polar ship formation navigation^59,60^. It is anticipated that the frequency of such incidents will decrease significantly in response to the reduced deployment of icebreakers, a trend that is projected to continue under various climate change scenarios. Furthermore, the risk associated with complex navigational conditions is expected to be substantially mitigated by the selection of the route proposed in this study, which avoids areas of narrow or shallow water depth.

## Data Availability

The data used in this research is from the Community Earth System Model 2 (CESM2) as part of the Coupled Model Intercomparison Project Phase 6 (CMIP6). The CESM2 data can be obtained from the official CESM website at https://www.cesm.ucar.edu/models/cesm2. Researchers interested in accessing the data are encouraged to follow the data distribution policies and terms of use specified on the CESM website.
